# SUBSTANTIAL EXTERNAL DOSE RATE VARIABILITY OBSERVED IN A COHORT OF LU-177 PATIENTS INDEPENDENT OF BMI AND SEX

**DOI:** 10.1093/rpd/ncac187

**Published:** 2022-09-22

**Authors:** Michael Bellamy, Bae Chu, Brian Serencsits, Brian Quinn, K Prasad, J Altamirano, Matthew Williamson, Daniel Miodownik, Natalie Abrahams, Fanny Chen, David Bierman, M Wutkowski, Lawrence Dauer

**Affiliations:** Department of Medical Physics, Memorial Sloan Kettering, 1275 York Avenue, New York, NY 10065, USA; Department of Medical Physics, Memorial Sloan Kettering, 1275 York Avenue, New York, NY 10065, USA; Department of Medical Physics, Memorial Sloan Kettering, 1275 York Avenue, New York, NY 10065, USA; Department of Medical Physics, Memorial Sloan Kettering, 1275 York Avenue, New York, NY 10065, USA; Department of Medical Physics, Memorial Sloan Kettering, 1275 York Avenue, New York, NY 10065, USA; Department of Medical Physics, Memorial Sloan Kettering, 1275 York Avenue, New York, NY 10065, USA; Department of Medical Physics, Memorial Sloan Kettering, 1275 York Avenue, New York, NY 10065, USA; Department of Medical Physics, Memorial Sloan Kettering, 1275 York Avenue, New York, NY 10065, USA; Siena College, 515 Loudon Road, Loudonville, New York, NY 12211, USA; Department of Medical Physics, Memorial Sloan Kettering, 1275 York Avenue, New York, NY 10065, USA; Department of Medical Physics, Memorial Sloan Kettering, 1275 York Avenue, New York, NY 10065, USA; Department of Medical Physics, Memorial Sloan Kettering, 1275 York Avenue, New York, NY 10065, USA; Department of Medical Physics, Memorial Sloan Kettering, 1275 York Avenue, New York, NY 10065, USA

## Abstract

External dose rates were measured 1 m away from 230 Lu-177 patients to characterise the variability in normalised dose rates as a function of administered activity, body mass index (BMI) and sex. The largest dose rate observed was 0.07 mSv/h associated with an administered activity of 7.2 GBq. Substantial variability was found in the distribution of the normalised dose rate associated that had an average of 0.0037 mSv/h per GBq and a 95% confidence interval of 0.0024–0.0058 mSv/h per GBq. Based on this study, estimating the patient dose rate based on the Lu-177 gamma exposure factor overestimates the dose rate by a factor of 2. A statistically significant inverse relationship was found between the patient dose rate and patient BMI and an empirically derived equation relating these two quantities was reported. On average, male patient dose rates were 3.5% lower than female dose rates, which may be attributed to the larger average BMI of the male patient group.

## INTRODUCTION

Lutetium-177 (Lu-177), in combination with sophisticated molecular carriers, is used extensively in hospitals for targeted radionuclide therapy in an outpatient setting^(1–4)^. Radiation doses to staff and members of the public need to be estimated, limited and optimised^(5)^ for this radiopharmaceutical to be used responsibly in the context of occupational and public health. US Nuclear Regulatory Commission (NRC) regulations permit a hospital to release a patient from its control if the total effective dose equivalent to a member of the public from the exposure to the patient is not likely to exceed 5 mSv per year. Additionally, the hospital must provide instructions to the patient if the total effective dose equivalent to a member of the public from the exposure to the patient is likely to exceed 1 mSv^(6, 7)^. To facilitate compliance with these regulations, the NRC provides guidance^(8)^ that describes the methods hospitals may use to determine whether the release of a patient administered with radioactive materials may be authorised. Under this guide, patients may be released based upon a threshold of the administered activity, the measured dose rate or a patient-specific dose calculation. Therefore, in this regulatory environment, it is valuable to characterise the distribution of administered Lu-177 activities, the associated external dose rate 1 m from the patient and any associated effect of a patient’s body mass index (BMI)^(9–11)^.

The dose rate near the patient is typically measured before a planned release to ensure that members of the public are not exposed to doses above the regulatory limit. One exception to this practice is where the administered activity is below a threshold. Otherwise, the absorbed dose to a member of the public can be estimated based on the initial dose rate and the time spent near the patient during biological retention and physical clearance^(12)^. If the initial dose rate or the biological retention is not known, these values can be substituted with conservative estimates such as the gamma exposure constant and the radionuclide’s physical half-life^(13)^. Such a conservative estimate would result in an overestimate to the dose rate 1 m from the patient and may result in expensive, inconvenient and unnecessarily long inpatient hospital stays^(14)^. On the other hand, a patient-specific calculation takes into consideration the patient’s actual dose rate and, if provided, their biological retention of radionuclide to determine the most accurate estimate of the patient’s post-administration dose rate, which, in turn, may allow for patient-specific instructions and the timely discharge of radionuclide patients^(15)^.

It is important to understand the distribution of dose rates associated with a particular administered activity. A limited number of peer-reviewed publications have examined the dose rates associated with Lu-177 administrations. Levart *et al*.^(16)^ calculated the equivalent dose to close relatives and colleagues of patients. Kurth *et al.*^(17)^ published initial and subsequent dose rates associated with 50 Lu-177 patients. These studies mainly focused on the effective half-life and integrated dose to nearby receptors with minimal focus on the estimation of initial dose rates. One notable exception is a paper published by Fitschen *et al.*^(18)^ where the dose rate distribution associated with 41 patient has been reported. At Memorial Sloan Kettering, Lu-177 has been routinely administered as an outpatient procedure for over 800 patients in accordance with the US Nuclear Regulatory Guidance. The goal of this study is to determine the distribution of initial dose rates associated with a large cohort of Lu-177 patients as a function of the administered activity, BMI and sex.

## METHODS

### Physical characteristics of Lu-177

Lu-177 decays to Hf-177 via beta-emission with a physical half-life of 6.647 d and a *Q*-value of 0.4983 MeV; these favourable decay characteristics make this radionuclide functional both for cancer therapy and dosimetric imaging^(19, 20)^. It is primarily produced in nuclear reactors via direct neutron capture by Lu-176 nuclei or indirect capture by Yb-176 nuclei. The primary gamma emission energies are 0.208 and 0.113 MeV. Lu-177 has a maximum beta spectrum energy of 0.4978 MeV and an average beta energy of 0.01333 MeV^(21)^. Based on the emission energies and probabilities, the gamma exposure constant associated with Lu-177 is 7.636E-6 mSv/hr per MBq (0.007636 mSv/hr per GBq) at 1 m^(22)^. While the gamma radiation allows the determination of the spatial distribution of Lu-177 after administration, it also contributes to the external exposure to staff, caretakers and members of the public. The beta emissions have a relatively short range and are primarily responsible for providing large radiation doses to the site of the tumour with a negligible effect on external dose rates^(23)^.

### Patients and Lu-177 administration

This study is based on post-administration dose rate measurements of 108 male and 122 female patients and presents a retrospective summary of initial Lu-177 whole-body dose rate readings. A total of 230 patient dose rates were included in the present analysis. Administered activities ranged from 3.59 to 7.91 GBq with an average activity of 6.90 GBq. These activities were administered by intravenous infusion over a period of approximately 1 hr with the associated measured dose rate taken shortly after completed administration. Dose rate measurements were taken and recorded in compliance with federal, state and city regulations associated with the release of medical patients. Standard medical radiation safety practices were employed throughout the treatment process including regular training of radiation workers, lead lined therapy rooms and contamination control protocols.

### Measurements

Whole body exposure rates were measured using an ionising chamber survey meter following the completion of the Lu-177 infusion. Exposure rates were measured using an auto-ranging 451B ionisation chamber survey meter developed by Fluke Biomedical with manufacturer-specified precisions of < 10%. In this study, the measured exposure rates were assumed to be numerically equal to absorbed dose rates at the point of measurement and all dose rate measurements mentioned in this paper^(13)^.

Exposure rates were measured using an ionisation chamber held at an anterior distance of 1 m from the torso of the patient. Typically, patients were seated in a reclined chair when the measurements were taken. Initial measurements were typically taken prior to patients’ voiding. On rare occasions, patients were permitted to void before the dose rate measurement to minimise the probability of a contaminated urine spill.

The ionisation chamber survey meters were calibrated annually in compliance with US federal regulations using ^137^Cs. No calibration correction was applied to the survey meter measurements because the energy response of Lu-177 (0.208- and 0.113-MeV photons) was assumed to be nearly identical to that of Cs-137 based on the manufacturer response curve^(24)^. Patient height and body weight were measured upon admission to the hospital.

### Data analysis

Univariate statistics were performed using Tableau 2020 Desktop Professional Edition, the Python SciPy statistics package^(25)^ and Microsoft Excel 365. This statistical analysis included the selection of the study population, determination of the mean, median and curve fitting to determine the relationship between the BMI and external radiation dose rate. BMI was calculated as the quotient of the patient mass (kg) and the square of patient height (m^2^). The activity normalised dose rate (referred to as normalised dose rate) was calculated as the quotient of the measured dose rate (mSv/h) and the administered Lu-177 activity (GBq) and represents the expected dose rate measurement if 1 GBq was administered to the patient. The line of best fit relating normalised dose rate to BMI was calculated using Tableau’s built-in analytics function. The exponential function was chosen because of the underlying physics of photon interaction with matter where the dose rate decreases exponentially with the thickness of shielding material.

## RESULTS

**Figure 1:**
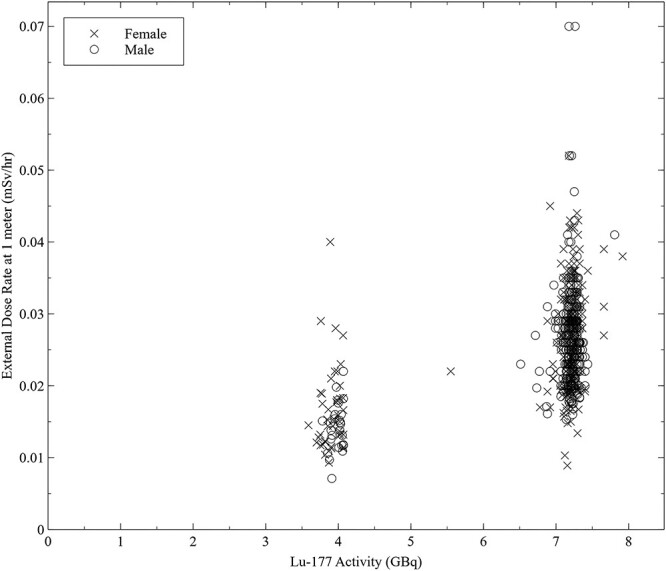
Initial external radiation dose rate measured at 1 m from a Lu-177 radiopharmaceutical patient as a function of administered activity. Administered activities are clustered around prescribed activities of 3.7 (100 mCi) and 7.4 GBq (200 mCi).

**Figure 2:**
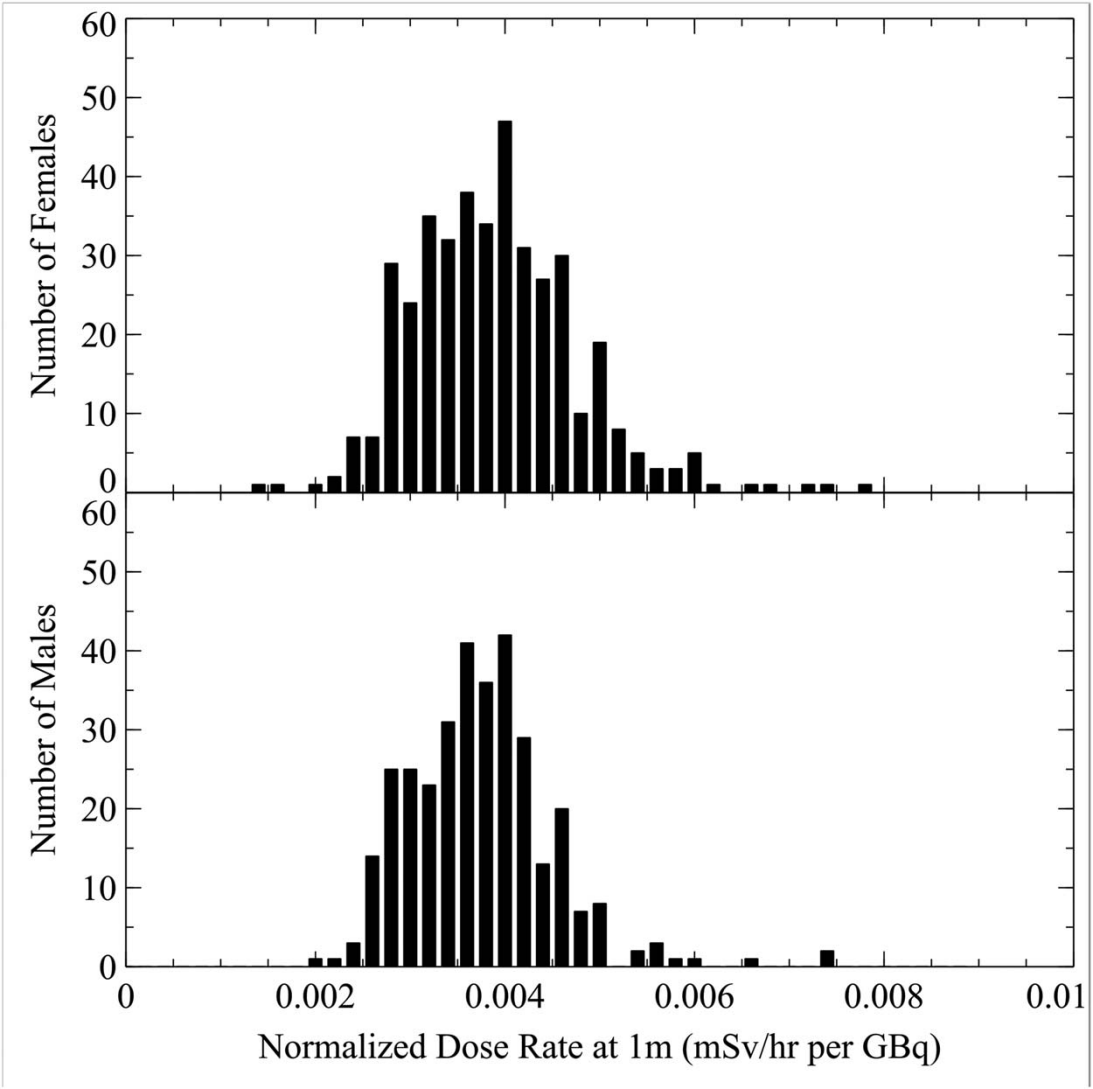
The histograms of sex-specific Lu-177 measurements as a function of activity-normalised dose rate measured at a distance 1 m (mSv/h per GBq). For reference, the gamma exposure constant for Lu-177 is 0.007636 mSv/hr per GBq.

**Figure 3:**
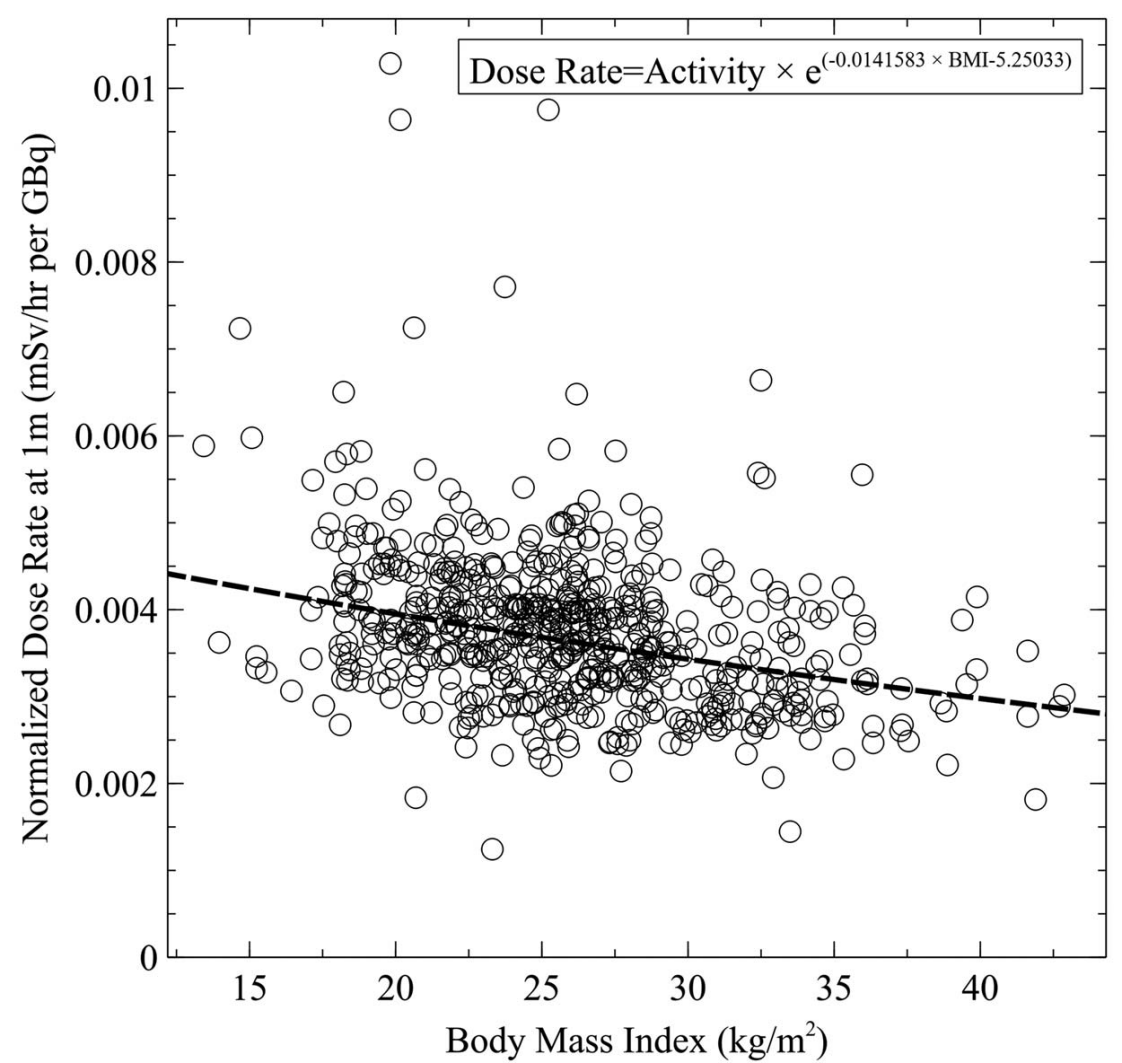
The relationship between Lu-177 normalised patient dose rate (mSv/h per GBq) at 1 m versus patient BMI (kg/m^2^) with a line of best fit. The included equation allows the total dose rate to be estimated based on the Lu-177 activity expressed in GBq and the patient BMI expressed in kg/m^2^.

Dose rate measurements associated with Lu-177 therapeutic administration were collected over a period of 5 y from 230 patients. Sex-specific dose rates with relation to Lu-177 activity are presented in Figure 1. Male and female patient activity-normalised dose rates measured at 1 m are shown in Figure 2. The relationship between the normalised dose rate and patient BMI is shown in Figure 3 with a line of best fit.

The ages of the patients ranged from 5 to 89 y old with an average age of 62.5 y old. Patient BMI ranged from 13.4 to 42.9 kg/m^2^ with an average of 25.9 kg/m^2^. Overall, the male patients had a higher BMI than female patients (27.2 for males versus 24.8 for females).

The overall average normalised dose rate associated with Lu-177 administration was found to be 0.0037 mSv/h per GBq with a 95% confidence interval of 0.0024–0.0058 mSv/h per GBq and a standard deviation of 0.000915 mSv/h per GBq, based on a normal statistical distribution. Median (50th), 2.5th and 97.5th percentile values for normalised dose rates were 0.0036, 0.0023 and 0.0049 mSv/h per GBq, respectively. Substantial variability was observed in the dose rate associated with fixed activities as shown in Figure 1. The largest dose rate observed was 0.07 mSv/h associated with an administered activity of 7.2 GBq. Administered activities ranged from 3.59 to 7.91 GBq with an average activity of 6.90 GBq. Most procedures were associated with an administered activity of 7.4 GBq (200 mCi). For females, the average normalised dose rate at 1 m was calculated to be 0.00380 mSv/h per GBq with a standard deviation of 0.0011 mSv/h per GBq. For male patients, the average normalised dose rate at 1 m was calculated to be 0.00367 mSv/h per GBq with a standard deviation of 0.001 mSv/h per GBq. Based on measurements from all patients in this study, Lu-177 dose rates (mSv/h) at 1 m can be estimated as a function of administered activity (GBq) and patient BMI (kg/m^2^) using equation 1.

(1) \begin{equation*} Dose\ Rate= Activity\times{e}^{-0.0141583\times BMI-5.25033} \end{equation*}

## DISCUSSION

The effects of using the conservative Lu-177 gamma exposure constant to estimate the dose rates 1 m from the patient can be seen in Figure 2. The gamma exposure constant for Lu-177 is 0.007636 mSv/hr per GBq, which is substantially higher than this study’s 95th percentile (0.0059 mSv/h per GBq). This implies that in the absence of measured patient dose rates, a suitable lower default than the gamma exposure factor could be used. Using the gamma exposure factor for members of the public dose calculations will result in a substantial overestimate akin to using only the physical half-life and ignoring biological removal. Despite the large variability observed in the patient population, the majority of the patients have a dose rate that is <50% of the gamma exposure constant. Specifically, the average normalised dose rate from the patient population was 48% of the Lu-177 point source gamma exposure constant.

Calais *et al.* discussed a successful outpatient radiopeptide therapy with the Lu-177 octreotate that targets neuroendocrine tumours in a study involving 76 patients with progressive metastatic tumours who received four doses of 7.8 GBq Lu-177 octreotate, with 8 weeks between each dose. The patient’s family members and caregivers were able to stay with the patient during treatment at the advised distance of 1 m and their exposure was monitored with an electronic dosemeter. The radiation exposure at 1 m from the patient after the infusion and before the patient voided was a mean rate of 34.9 μSv/hr with a range of 22.8 to 47.2 μSv/hr. Based on the stated administered activity, the normalised dose rate of 0.0044 mSv/h per GBq from this study is higher than our findings (0.0037 mSv/h per GBq). The published range of normalised dose rates of this smaller patient cohort is similar to the 95% confidence interval of the current study^(26)^. Similar findings were noted from comparisons with dose rates published by Levart *et al.*^(16)^.

A large degree of variability in the dose rate is evident from Figures 1, 2 and 3. For the same administered activity, some patient dose rate measurements ranged considerably from the average. This variability is not explained by the differences in administered activity as shown in the broad distribution of normalised dose rates of Figure 2. Additionally, this large variability exists independently both for males and females, although the standard deviation of the dose rate is larger in the female population. According to the manufacturer of the ionisation chamber meters, the uncertainty associated with the dosemeters was <10%, which also fails to account for the broad distribution of normalised dose rates.

Statistically significant differences in dose rates were noted between the dose rate distribution of the male patients versus the female patients. The average dose rate was 0.0037 and 0.0038 mSv/h per GBq for the male and female patients, respectively. A two-sample *z*-test was conducted for the two distributions denoted in Figure 2 demonstrated a statistically significant difference with a *p*-value of 0.004. Despite this statistical result, the close averages of the male and female groups indicates that patient sex has negligible predictive value in estimating initial dose rates for Lu-177. The observed difference could potentially be attributed to the larger average BMI of the malepatients (27.3 kg/m^2^) versus the female patients (24.7 kg/m^2^).

A significant association between the normalised dose rate and BMI was observed based on the entire patient population of this study. Spearman rank correlation was computed to assess the relationship between the normalised patient dose rate at 1 m and the corresponding patient BMI. There was a negative spearman rank correlation between the two variables *R* = −.35, *P* < 0.001. The strong association between BMI and dose rate is expected because patients with lower BMI would generally have less self-shielding as the average length of tissue between the Lu-177 decay and the detector would be shorter in a lower BMI patient. In this work, no association between patient BMI and the administered activity was observed^(27)^.

The primary limitation of this work stems from the reliance on the retrospective patient dose rate measurements collected for regulatory compliance. Before a radiotherapy patient is released, the exposure rate near to the patient must be measured and recorded. While patients were typically in a reclined position, there was variability in the patient geometry when the measurements were taken. That is, some patients were reclined, whereas others were lying down or sitting upright. The variation in patient geometry could be responsible for some of the observed exposure rate variability. In addition, occasionally patients wore blankets or covering that have a small attenuation effect on the measured exposure rates.

Another limitation comes from the distance at which the measurements are recorded. Although it is routine practice to use a tape measure to ensure that measurements were taken at exactly 1 m, small shifts in the position of the measuring tape or patient can affect the final exposure rate. While waiting for the reading on the exposure rate meter to stabilise, the exact location of the ionisation chamber can potentially shift by a few centimetres. Likewise, during that period, the patient can potentially move themselves to be either slightly closer or further away from the meter. Furthermore, the tumour burden could have an impact on the dose rate after radionuclide uptake. Therefore, the tumour’s geometric distribution within the torso could have an effect on the measured dose rates, which was not considered in this study. This effect is expected to be relatively small because the measurements were taken immediately after the Lu-177 infusion. Dose rate measurements at later timepoints may be significantly affected by the patient’s tumour burden.

Finally, the time at which the measurements were recorded was not accounted for in this study. In the vast majority of cases, measurements are taken immediately after the completion of the infusion that includes the flush. However, occasionally patients would void during or immediately post-administration. Because the patient voiding status is not noted, the measured exposure rates could be lower (16) than expected in these cases.

## CONCLUSION

External radiation dose rates at 1 m are summarised for 230 Lu-177 patients based on ionisation chamber survey measurements as a function of patient sex and BMI. The largest dose rate observed was 0.07 mSv/h associated with an administered activity of 7.2 GBq. Substantial variability was found in the distribution of normalised dose rate associated that had an average of 0.0037 mSv/h per GBq and a 95% confidence interval of 0.0024–0.0058 mSv/h per GBq. Based on the measurements of this study and the gamma exposure factor at 1 m, Lu-177 has an average patient self-shielding factor of 48%. A statistically significant inverse relationship was found between the patient dose rate and patient BMI, and an empirically derived equation was reported. Small, but statistically significant differences were found in the average normalised dose rate between males and females, which could potentially be a result of the larger average BMI of the male patients.

## FUNDING

The National Institutes of Health/National Cancer Institute Cancer Center (grant P30 Ca008748).

## CONFLICT OF INTEREST

The authors declare no conflict of interest.
